# Continuous Light-Induced PCOS-Like Changes in Reproduction, Metabolism, and Gut Microbiota in Sprague-Dawley Rats

**DOI:** 10.3389/fmicb.2019.03145

**Published:** 2020-01-21

**Authors:** Weiwei Chu, Junyu Zhai, Jieying Xu, Shang Li, Weiping Li, Zi-Jiang Chen, Yanzhi Du

**Affiliations:** ^1^Center for Reproductive Medicine, Renji Hospital, School of Medicine, Shanghai Jiao Tong University, Shanghai, China; ^2^Shanghai Key Laboratory for Assisted Reproduction and Reproductive Genetics, Shanghai, China; ^3^Center for Reproductive Medicine, Shandong University, Jinan, China; ^4^National Research Center for Assisted Reproductive Technology and Reproductive Genetics, Jinan, China; ^5^The Key Laboratory for Reproductive Endocrinology of the Ministry of Education, Center for Reproductive Medicine, Jinan, China

**Keywords:** circadian rhythm disorder, PCOS, reproduction, metabolism, microbiota

## Abstract

The interplay between genetic and environmental risk factors contributes to the pathogenesis of metabolic disease. Polycystic ovary syndrome (PCOS) is the most common endocrine and metabolic disorder in women of reproductive age. Circadian rhythm disruption is an important risk factor for PCOS. In this study, we evaluated the effect of circadian disorder on reproduction as well as metabolism, and determined its influence on gut microbiota in a rat model. Female Sprague Dawley (SD) rats were kept under continuous light exposure (12-h:12-h light/light cycle, L/L group) or a control cycle (12-h:12-h light/dark cycle, L/D group) for four consecutive weeks. Manifestations in endocrine hormones and metabolism were detected and gut microbiota were analyzed with the 16s rRNA gene sequencing technique. To our knowledge, this is the first study to report PCOS-like reproductive manifestation, such as anti-Müllerian hormone (AMH) elevation induced by continuous light exposure. Moreover, continuous light resulted in abnormal glucose metabolism and gut microbial community variations, including enrichment of the microbial genus of *Parasutterella* and reduced abundance of genus *Corynebacterium*, genus *Odoribacter*, and genus *Acinetobacter.* Increased *Parasutterella* abundance was positively correlated with serum testosterone level. A PICRUSt analysis revealed that reproductive and metabolic-related genes were enriched in rats of L/D group. In conclusion, the present study demonstrates that continuous light exposure, an important environmental factor, contributes to the occurrence and developmental progress of PCOS and changes in microbial component and structure. Continuous light exposure is one of vital causes of PCOS, which is closely related to microbial structure and functions.

## Introduction

Polycystic ovary syndrome (PCOS) is the most common endocrine disorder in women of reproductive age, with an estimated prevalence of approximately 10 to 15% worldwide (Azziz et al., 2016). According to criteria of the National Institutes of Health (NIH), PCOS patients present with the combination of chronic oligo- or anovulation, clinical or biochemical signs of hyperandrogenism, and ovary enlargement (size >10 mL) or 12 or more small follicles (2–9 mm) under ultrasound without related disorders (Rotterdam Eshre/Asrm-Sponsored Pcos Consensus Workshop Group, 2004). Women with PCOS also have accompanying metabolic disorders, such as obesity, insulin resistance, and type 2 diabetes mellitus (El-Mazny et al., 2010). A variety of studies have shown that genetic abnormalities, lifestyle, prenatal hormonal imbalance, and environmental factors may contribute to PCOS (Vink et al., 2006; Wen et al., 2019). However, the cause of this syndrome remains obscure.

Among the environmental factors that may contribute to PCOS, circadian rhythm disruption has recently received significant interest (Simon et al., 2019). In cases where the timing of the light-dark cycle altered as a result of transmeridian flight or shift work, the circadian system frequently is misaligned with the external physical environment, leading to poor health outcomes (Farhud and Aryan, 2018). Several studies have shown that women exposed to night-light shifts had irregular menstrual cycles (Lawson et al., 2011) that were frequently associated with dysmenorrhea and metabolic syndrome (Lim et al., 2018), insulin resistance, and deregulation of glucose metabolism (Sharma et al., 2017), all of which are recognized risk factors for PCOS (Ali, 2015). Prolonged light exposure was reported to induce polycystic ovaries and hyperandrogenism in rodents as well (Kang et al., 2015, 2017; Paixão et al., 2017), indicating the relationship between circadian rhythm disorder and manifestation of PCOS. However, the effect of circadian rhythm disruption on reproductive hallmarks of PCOS and the mechanism underlying this pathogenesis remain unclear. Clarifying these issues could help to understand the occurrence and development of PCOS in women with circadian rhythm disturbance.

Gut flora are vital in the host physiologic process (Velagapudi et al., 2010). Changes in gut microbial composition may be unfavorable and lead to an individual being predisposed to disease. The advent of new molecular techniques, such as 16s rRNA sequencing, has made it possible to identify the gut microbiome composition and reveal the relationship between gut flora and metabolic disease (Cani et al., 2008). Accumulating studies have reported the association between microbiota and PCOS in human (Lindheim et al., 2017; Liu et al., 2017; Insenser et al., 2018) and rodent models (Guo et al., 2016; Noroozzadeh et al., 2017). In women with PCOS, α-diversity was decreased and the phylogenetic profile of gut microbiota was different (Lindheim et al., 2017). Many scientists found that bacteria belonging to *Bacteroides, Escherichia/Shigella* and *Streptococcus*, were positively correlated with clinical reproductive parameters, such as testosterone and BMI (Liu et al., 2017; Noroozzadeh et al., 2017). Another study revealed the possible role of gut microbiota in the pathology of PCOS (Guo et al., 2016). It claimed that treatment with *Lactobacillus* or fecal microbiota transplantation from healthy subjects ameliorated estrous cycle disorders and hyperandrogenism in PCOS rat models. The imbalanced gut microbiota in PCOS patients and its association with clinical reproductive parameters suggest the importance of gut microbiota in PCOS manifestation. Altered gut flora may be associated with the occurrence of PCOS and make a contribution to its manifestation.

Although some studies uncovered probable causes of PCOS, the role of circadian rhythm disorder in its etiology remained to be explained. To determine the effects of rhythm disturbance on the presentation of PCOS, we applied four-week continuous light exposure in Sprague Dawley (SD) rats and examined their reproductive and metabolic manifestations. Additionally, to determine the role of gut microbiota, we collected the fecal samples of SD rats and employed high-throughput sequencing of the bacterial 16S rRNA genes. The changes in reproduction, metabolism, and gut microbial constitution under influence of continuous light were evaluated. The results of this study provide new insights into the pathologic mechanism of PCOS occurrence and offer basic research evidence for clinical diagnosis and treatment in reproductive women suffering from circadian rhythm disturbance.

## Materials and Methods

### Animals and Treatments

Female SD rats (200–220 g) of 6 weeks were purchased from the China National Laboratory Animal Resource Center (Shanghai, China) and randomly divided into two groups: control group and light group (*N* = 11/group). The control group was under a periodic cycle (12-h/12-h light/dark cycle, L/D group) while the light group was exposed to continuous light (12-h/12-h light/light cycle, L/L group) for four consecutive weeks. All rats were housed in a temperature-controlled room (22–24°C) under specific pathogen-free conditions. Rats were allowed to adapt to the new environment for 1 week before our experiments with access to standard chow and demineralized water *ad libitum*.

Body weight (BW) was monitored once a week throughout the experiment and estrous cycles were monitored daily by exfoliative cytoscopy during the last 6 days. An intraperitoneal glucose tolerance test (IPGTT) was performed at 9 a.m the day before the rats were sacrificed. At the termination of the experiment, all rats were deeply anesthetized to sacrificed, serum, bilateral ovaries, parametrial adipose tissue, muscle, and liver were collected. The L/D group and L/L group were sacrificed at the diestrus stage of the estrous cycle.

All experimental protocols were performed in accordance with institutional guidelines. The study was approved by the research committee of the Shanghai Jiao Tong University School of Medicine (number: 2016-1220).

### IPGTT

All SD rats were fasted overnight for 16 h (5 p.m. to 9 a.m.) followed by i.p. injection of 50% D-glucose normal saline solution (China Otsuka Pharmaceutical Co., Ltd., China) at 2 g/kg body weight. Blood samples for glucose measurements were obtained from the tail vein at the indicated times with an Accu-Chek glucose monitor (Roche, Basel, Switzerland).

### Hormone Profile and Insulin Resistance Index

Trunk circulation blood samples were obtained and allowed to incubate for 4 h at room temperature. All samples were centrifuged (2,500 rpm, 15 min, 4°C) and serum was collected into 1.5 ml EP tubes and kept at −80°C for subsequent experiments.

Total serum testosterone and estradiol were measured using ELISA kits from Cayman Chemical (Ann Arbor, MI, United States). Serum corticosterone and leptin were measured with ELISA kits from R&D Systems, Inc. (Minneapolis, MN, United States). The levels of anti-Müllerian hormone (AMH), follicle stimulating hormone (FSH), luteinizing hormone (LH), thyroid stimulating hormone (TSH), progesterone, and insulin were measured using ELISA kits from CUSABIO Technology LLC (United States), Aviva Systems Biology, Corp. (United States), Enzo Life Sciences (United States), Uscn Life Science Inc. (Belgium), TSZ biological Trade Co., Ltd. (United States), and MyBioSource, Inc. (San Diego, CA, United States), respectively. The sensitivity of ELISA kits of insulin, LH, FSH, estradiol, testosterone, corticosterone, leptin, progesterone, TSH, and AMH were lower than 0.312 μIU/mL, 5.2 mIU/mL, 0.156 mIU/mL, 15 pg/mL, 6 pg/mL, 0.028 ng/mL, 22 pg/mL, 15 pmol/L, 0.3 μIU/mL, and 0.1 ng/mL, respectively. The coefficient of variation of the kits mentioned above were 6.7%, 3.9%, 5.9%, 11.9%, 6.0%, 4.5%, 3.3%, 10%, 15%, and 15%, respectively. All ELISAs were used to detect indexes in rats and performed according to the manufacturer’s instructions.

The homeostasis model assessment of insulin resistance (HOMA-IR) and homeostasis assessment of β-cell function (HOMA-β) were calculated for each rat using the following equations (Shawky et al., 2019):

HOMA-IR=[fastingbloodglucose(mmol/L)

×fastinginsulin(mIU/L)]/22.5

HOMA-β=20×fastinginsulin(FINS,mIU/L)/

(fastingplasmaglucose(mmol/L)-3.5)(%)

### Vaginal Smears

The stage of the estrus cycle was determined by the predominant cell type in vaginal smears that were obtained from 9 weeks of age until the end of the experiment (Marcondes et al., 2002).

### Histology

Rat ovarian tissues were fixed with 4% paraformaldehyde, and then embedded in paraffin. Serial sections of 5 μm-thick ovarian tissue sections were deparaffinized and rehydrated through a graded ethanol series. Then the sections were stained in hematoxylin and differentiated by hydrochloric acid. Finally, the sections were incubated in eosin before covering the slides or visualization using a microscope (Zeiss, Oberkochen, Germany). Total numbers of small antral follicles (oocyte surrounded by greater than five layers of granulosa cells and/or one or two small areas of follicular fluid), large antral follicles (containing a single large antrum), atretic cyst-like follicles (large fluid-filled cysts with an attenuated granulosa cell layer, dispersed theca cell layer, and an oocyte lacking connection to the granulosa cells) and corpora lutea (CL) were classified and quantified as previously reported (Caldwell et al., 2017). To avoid repetitive counting, each follicle was only counted in the section where the oocyte’s nucleolus was visible.

Parametrial fat pads were fixed in 4% paraformaldehyde, embedded in paraffin, and sectioned at 8 μm. Sections were stained with hematoxylin and eosin before images were taken for histomorphometry at 20 × magnification under a light microscope. Five distinct images were taken from each of three sections of the fat pad, with at least 200 μm separating these sections.

### Detection of Gene Expression Using Quantitative Reverse Transcriptase Polymerase Chain Reaction (qRT-PCR)

Liver, parametrial adipose tissue, and muscle samples were rapidly dissected, snap-frozen in liquid nitrogen, and stored at −80°C for RNA extraction. Total RNA was extracted from these specimens using a total RNA kit following the manufacturer’s instructions (FOREGENE, Chengdu, China). RNA concentration and quality were determined by measuring optical density at 260 nm (OD260) and the ratio of OD260/OD280 with NanoDrop ND-2000, and electrophoresis on a denaturing agarose gel. The mRNA from the total cellular RNA was reverse-transcribed to cDNA using PrimeScript RT Master Mix Perfect Real Time kit (TaKaRa, Dalian, China). 500 ng of RNA were used in reverse transcription and qRT-PCR was measured using the following settings: 2 min pre-incubation at 94°C followed by 35 cycles of denaturation at 94°C for 30 s, annealing at 61°C for 20 s, elongation at 72°C for 20 s. Fluorescence was measured at the end of the elongation phase using 470 nm excitation and 530 nm emission (channel 1). Correct PCR products were confirmed by melting curve analysis. The absolute mRNA in each sample was calculated according to a standard curve setup using serial dilutions of known amounts of specific templates against corresponding cycle threshold (Ct) values (Gadkar and Filion, 2014). The housekeeping gene β*-actin* was amplified in parallel as an internal loading control. The primer sequences of targeting genes used were described in Supplementary Table S1. The ratio of the target gene over β*-actin* was obtained as the target mRNA levels.

### Microbial Diversity Analysis

#### Sample Collection

Five female rats were selected randomly from L/D group and L/L group, respectively. Fresh fecal samples were taken from these rats once a week throughout the experiment, collected into 1.5 ml sterile EP tubes, rapidly snap-frozen in liquid nitrogen, and stored at −80°C until further analysis.

#### Fecal DNA Extraction and PCR Amplification

Microbial DNA was extracted from fecal samples using the QIAamp Fast DNA Stool Mini Kit (Qiagen, Valencia, CA, United States), according to manufacturer’s protocols. The V3-V4 region of the bacteria 16S ribosomal RNA genes was amplified by PCR (95°C for 3 min, followed by 30 cycles at 98°C for 20 s, 58°C for 15 s, and 72°C for 20 s, with a final extension at 72°C for 5 min) using primers 341F 5′-CCTACGGGRSGCAGCAG)-3′ and 806R 5′-GGACTACVVGGGTATCTAATC-3′. PCR reactions were performed in 30 μL mixture containing 15 μL 2 × KAPA Library Amplification ReadyMix, 1 μL of each primer (10 μM), 50 ng of template DNA, and ddH_2_O.

#### Illumina MiSeq PE250 Sequencing

Amplicons were extracted from 2% agarose gels and purified using the AxyPrep DNA Gel Extraction Kit (Axygen Biosciences, Union City, CA, United States), according to the manufacturer’s instructions, and were quantified using Qubit^®^ 2.0 (Invitrogen, Carlsbad, CA, United States). After preparation of library, these tags were sequenced on the MiSeq platform (Illumina, Inc., San Diego, CA, United States) for paired end reads of 250 base pairs (bp), which were overlapped on their three ends for concatenation into original longer tags. DNA extraction, library construction, and sequencing were conducted at the Realbio Genomics Institute (Shanghai, China).

#### Sequencing Data Process

Tags, trimmed of barcodes and primers, were further checked on their rest lengths and average base quality. Tags were restricted between 220 and 500 bp, such that the average Phred score of bases was no worse than 20 (Q20) and no more than three ambiguous N (Masella et al., 2012). The copy number of tags was enumerated and redundancy of repeated tags was removed. Only tags with a frequency >1, which tended to be more reliable, were clustered into operational taxonomic units (OTUs), each of which had a representative tag (Edgar, 2013). OTUs were clustered with 97% similarity using UPARSE^1^ and chimeric sequences were identified and removed using Usearch (version 7.0). Each representative tag was assigned to a taxa by RDP Classifer^2^ against the RDP database^3^ using a confidence threshold of 0.8 OTU profiling table. To evaluate the community diversity and community richness, the Chao1 value was calculated using Mothur (version V.1.30.1; the University of Michigan, Ann Arbor, MI, United States). Principal components analysis (PCoA) of the OTUs in different groups was conducted using R package (version 3.1.0; R Foundation for Statistical Computing, Vienna, Austria) to compare the constitution of gut microbiota. LEfSe analysis coupled with the Kruskal-Wallis rank sum test was performed to identify the microbial differences among all groups. The linear discriminant analysis (LDA) score for each bacterium was calculated and a taxonomic cladogram was constructed to visualize the differences in microbiol composition. Predictive function analysis was performed using the PICRUSt algorithm based on the Kyoto Encyclopedia of Genes and Genomes Orthology (KO) classification (Langille et al., 2013). Sequencing data were submitted to the NCBI Sequence Read Archive under Accession PRJNA508095.

### Statistical Analysis

All data are represented as mean ± standard error of the man (SEM). After examination for normal distribution, data were analyzed using a paired Student’s *t*-test or one-way analysis of variance test followed by the Newman–Keuls multiple comparison test, where appropriate, to assess significant differences. In LEfSe analysis, a significant value of <0.05 and LDA effect size of >2 were used as thresholds. Significance was shown as ^∗^*P* < 0.05, ^∗∗^*P* < 0.01, or ^∗∗∗^*P* < 0.001.

## Results

### Continuous Light-Induced PCOS-Like Changes in SD Rats

**FIGURE 1:**
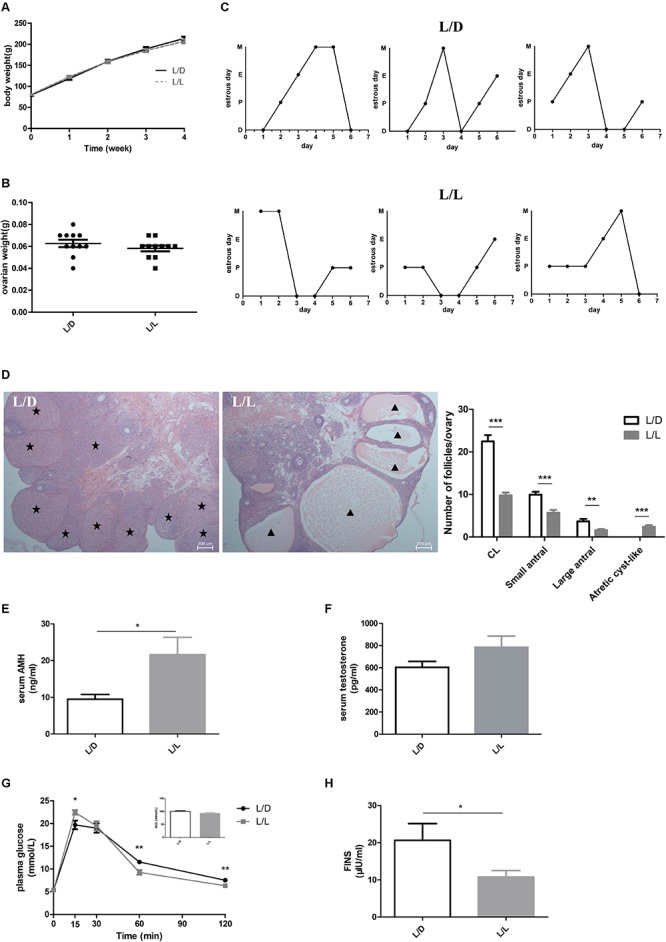
Continuous light-induced PCOS-like changes in SD rats. **(A)** The average weekly BW of each group of rats. **(B)** The ovarian weight of each group of rats. **(C)** Representative estrous cycles of two groups. The upper and lower panels represent the L/D and L/L groups, respectively. D, diestrus; P, proestrus; E, estrus; M, metestrus. **(D)** Representative examples of ovarian HE-stain histology and average numbers of follicles and CL per ovary in L/D and L/L group. **(E)** Serum AMH concentration. **(F)** Serum T concentration. **(G)** Glucose tolerance test and the areas under the curves. **(H)** Fasting insulin level. Star, CLs; triangle, arrested antral follicle. CL, corpora lutea. *N* = 11/group. Values are expressed as means ± SEM. Significant differences between the two groups are indicated by asterisks (^∗^*P* < 0.05, ^∗∗^*P* < 0.01, ^∗∗∗^*P* < 0.001).

After experimental treatment for 4 weeks, there was no difference in BW or ovarian weight between the L/L and L/D groups (Figures 1A,B; *P* > 0.05). The L/L group of rats had prolonged estrous cycles (Figure 1C) and the incidence of disordered estrous cycle was higher in the L/L group (Table 1, *P* < 0.05). Histologically, the number of CL present was notably diminished in L/L group, demonstrating oligo/anovulation (Figure 1D, *P* < 0.001). The number of small antral follicles as well as large ones significantly decreased (Figure 1D; *P* < 0.001 and *P* < 0.01, respectively). Additionally, as a previous study reported (Paixão et al., 2017), we observed more abundant atretic cyst-like follicles in the ovaries of L/L group (Figure 1D, *P* < 0.001). Similarly, oligo/anovulation is a key reproductive characteristic of PCOS.

**TABLE 1:** Incidence of estrous cycle disorder in the L/D and L/L groups.

**Group**	**No. of rats with ECDs**	**No. of rats without ECDs**	**Total**	**Incidence**	***P* value**
L/D	2	8	10	20%	0.023
L/L	8	2	10	80%	
Total	10	10	20	–	

*ECD, estrous cycle disorder. Results are expressed as mean ± SEM.*

To explore the influence of continuous light on endocrine system, we measured the concentrations of important hormones in the two groups. Firstly, we found serum AMH level was clearly increased (Figure 1E, *P* < 0.05), while the difference in testosterone abundance between the two groups was not significant (Figure 1F, *P* > 0.05). There were no differences in concentrations of LH, FSH, estradiol, progesterone, or TSH between the two groups (Supplementary Figures S1A–E, respectively; *P* > 0.05). Moreover, to investigate the effect of stress on endocrine system induced by continuous light exposure, we measured one of the factors, the corticosterone level and found no significant difference between the two groups (Supplementary Figure S1F, *P* > 0.05).

Despite the abnormal reproductive manifestation, we found metabolic disturbance in L/L group of rats. In glucose tolerance test (Figure 1G), plasma glucose level at 15 min was much higher in L/L group (*P* < 0.05), and dropped much lower at 60 min and 120 min compared to L/D group (*P* < 0.01). Although the area under curve (AUC) was not different between two groups (Figure 1G, *P* > 0.05), abnormal glucose level and increased postprandial glycemia level indicated impaired glucose metabolism in L/L group. In addition, we found basal insulin concentration was significantly lower in L/L group of rats (Figure 1H, *P* < 0.05). Leptin, another hormone that plays key role in the regulation of metabolism, remained unchanged in L/L group (Supplementary Figure S1G, *P* > 0.05). The adipocyte size in L/L group was comparable to that of L/D group (Supplementary Figure S1H).

The phenotype induced by light exposure in SD rats included estrous cycle disorder, oligo/anovulation, ovulation dysfunction, elevated AMH level, and abnormal glucose metabolism, which was mostly consistent with manifestation of PCOS in patients (Bozdag et al., 2016). In this respect, continuous light for 4 weeks resulted in reproductive changes and glucose intolerance, conducting the PCOS-like model in SD rats successfully.

### Continuous Light Resulted in Impaired Glucose Metabolism in Liver, Adipose Tissue, and Muscle

**FIGURE 2:**
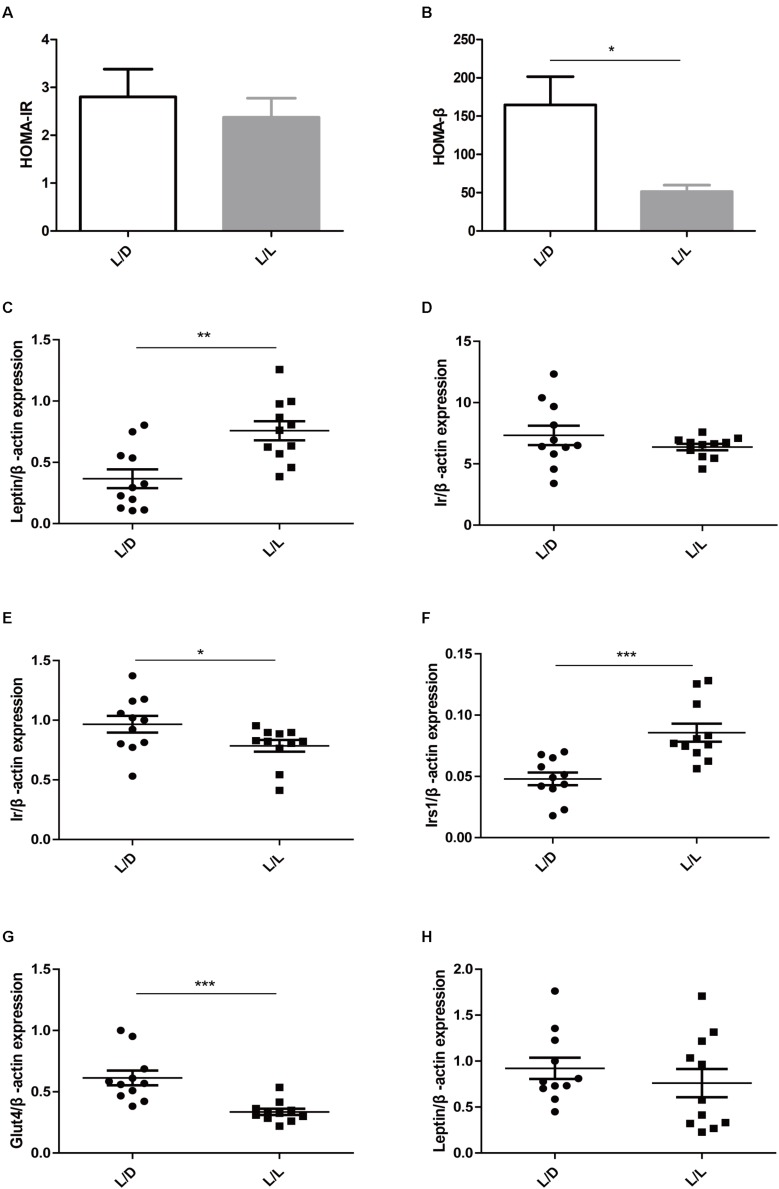
Continuous light resulted in impaired glucose metabolism in liver, adipose tissue, and muscle. **(A)** HOMA-IR. **(B)** HOMA-β. **(C,D)** The mRNA abundance of *Leptin* and *Ir* in liver tissue, respectively. **(E,F)** The mRNA abundance of *Ir* and *Irs1* in adipose tissue, respectively. **(G,H)** The mRNA abundance of *Glut4* and *Leptin* in muscle, respectively. *N* = 11/group. Values are expressed as means ± SEM. Significant differences between the two groups are indicated by asterisks (^∗^*P* < 0.05, ^∗∗^*P* < 0.01, ^∗∗∗^*P* < 0.001).

To learn more about metabolic abnormality in light-induced PCOS rats, we conducted homeostasis model assessment including HOMA-IR and HOMA-β in the two groups. HOMA-IR is a valid measure to determine insulin-resistance in rat model (Antunes et al., 2016), while HOMA-β is a widely used clinical and epidemiological tool for the assessment of β−cell function (Matthews et al., 1985). In this study, although HOMA-IR was identical (Figure 2A, *P* > 0.05), the HOMA-β was obviously lower in L/L group of rats (Figure 2B, *P* < 0.05), indicating poor β−cell function after continuous light exposure.

Then we detected the expression level of the genes that were closely relevant with glucose metabolism. Insulin receptor, insulin receptor substrate 1, leptin, and glucose transporter type 4 were reportedly associated with glucose metabolism in PCOS (He et al., 2019). After four-week constant illumination, the *Leptin* mRNA expression levels were significantly higher (Figure 2C, *P* < 0.01), while the expression levels of *Ir* (*insulin receptor*) remained unaffected in liver tissue (Figure 2D, *P* > 0.05). Additionally, continuous light treatment reduced the relative expression of *Ir* (Figure 2E, *P* < 0.05), but elevated the expression level of *Irs1* (*insulin receptor substrate 1*) in adipose tissue (Figure 2F, *P* < 0.001). In muscle of L/L group, the mRNA abundance of *Glut4* (*glucose transporter type 4*) reduced significantly (Figure 2G, *P* < 0.001), while expression of *Leptin* remained unchanged (Figure 2H, *P* > 0.05). These data suggested poor β−cell function and impaired insulin signaling peripherally might contribute to lower basal insulin level and abnormal glucose metabolism. Continuous light exposure for 4 weeks resulted in glucose metabolic abnormality in PCOS rats.

### Time-Dependent Change of Gut Microbiota Revealed a Possible Link Between Continuous Light and Metabolic Disorder of PCOS

**FIGURE 3:**
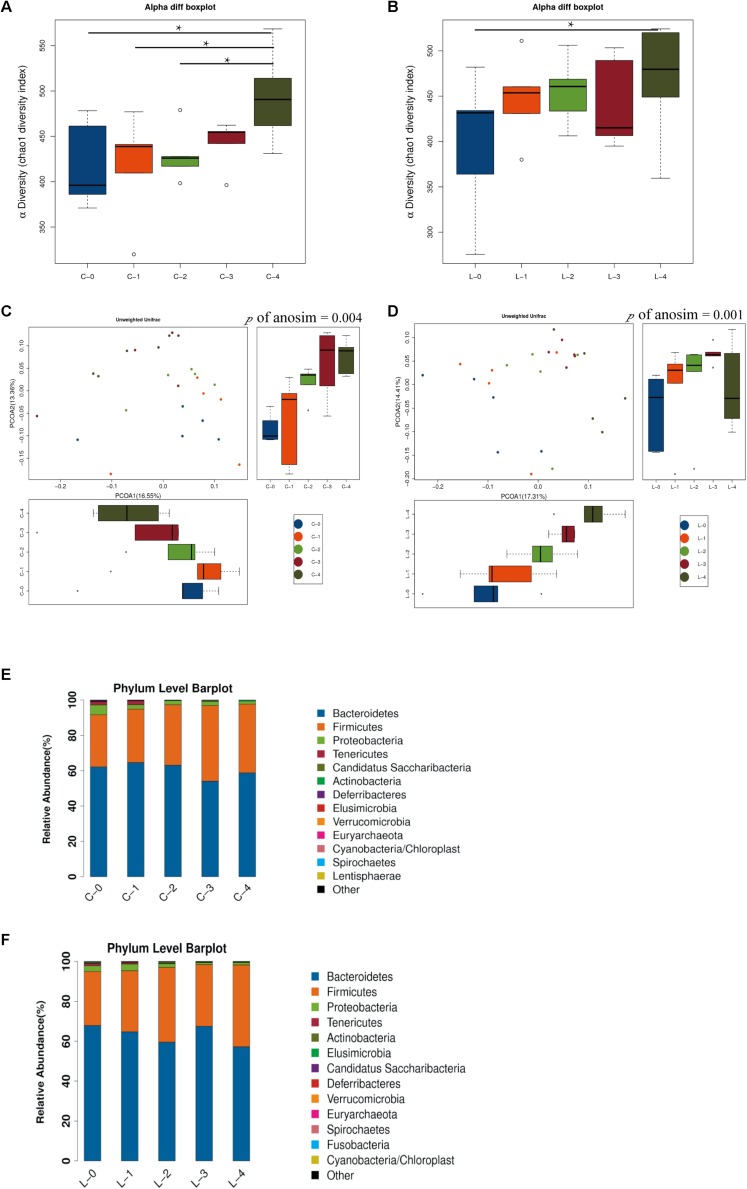
Time-dependent alterations of gut microbiota community in L/D group and L/L group. **(A,B)** Boxplots of α-diversity (Chao1 diversity index) among time points in the L/D and L/L groups, respectively. **(C,D)** PCoA plot with different relative abundances of OTUs among time points in the L/D and L/L groups, respectively. **(E,F)** Taxonomic compositions of bacterial communities in phylum level among time points in the two groups. C-0, C-1, C-2, C-3, C-4 indicated the clusters of samples collected at the weekly time point in the L/D group of rats, respectively. L-0, L-1, L-2, L-3, L-4 indicated the clusters of samples collected at the weekly time point in the L/L group of rats. *N* = 5/group. One-way ANOSIM analysis was performed to show the differences among time points. Significant differences between groups were indicated by *P* < 0.05.

To investigate the changes in gut microbiota as time went by, we compared the gut microbiota among different weeks. Chao1 index showed the alpha diversities of rats were significantly higher in week 4 compared with weeks 0, 1, and 2 in L/D group (Figure 3A, *P* < 0.05). In the L/L group, alpha diversity was increased in rats in week 4 compared with that of week 0 (Figure 3B, *P* < 0.05). Besides, the bacterial communities of rats in different weeks were clearly separated from each other by PCoA based on unweighted Unifrac in L/D group (Figure 3C) as well as in the L/L group (Figure 3D). Differences in community membership among different weeks were significant by ANOSIM in L/D (*R* = 0.217, *P* < 0.01) and L/L group (*R* = 0.276, *P* < 0.01).

The community compositions of L/D and L/L group showed that two groups of bacteria were dominant in the gut microbiota of SD rats: *Bacteroidetes* and *Firmicutes* (Figures 3E,F, respectively). The relative abundance of these two predominant microbial divisions (phylum level) differed among weeks in L/L group of rats: rats under continuous light exposure for 4 weeks had more *Firmicutes* and fewer *Bacteroidetes* than those at the beginning of experiment (Table 2). On the contrary, the relative abundance of *Firmicutes* and *Bacteroidetes* were unaffected between week 0 and week 4 in the L/D group. The abundance alterations in *Firmicutes* and *Bacteroidetes* in L/L group of rats were in accordance with the changes in metabolic disorder (Wang H. et al., 2018). This alteration in a time-dependent manner reflected the association between continuous light and metabolic disturbance. Despite the alterations in microbiota along with the ages of rats, continuous light could result in an abnormal gut profile resembling metabolic disorder. Based on these findings, we supposed that gut microbiota imbalance might function as a link of circadian disturbance and glucose intolerance in PCOS rats.

**TABLE 2:** Firmicutes/Bacteroidetes ratio at different experimental stages.

**F/B ratio**	**week 0**	**week 4**	***P* value**
L/D	0.56 ± 0.18	0.68 ± 0.07	0.46
L/L	0.41 ± 0.07	0.75 ± 0.13	0.05

*Results are expressed as mean ± SEM.*

### Gut Microbiota Altered After Continuous Light Exposure for 4 Weeks in SD Rats

**FIGURE 4:**
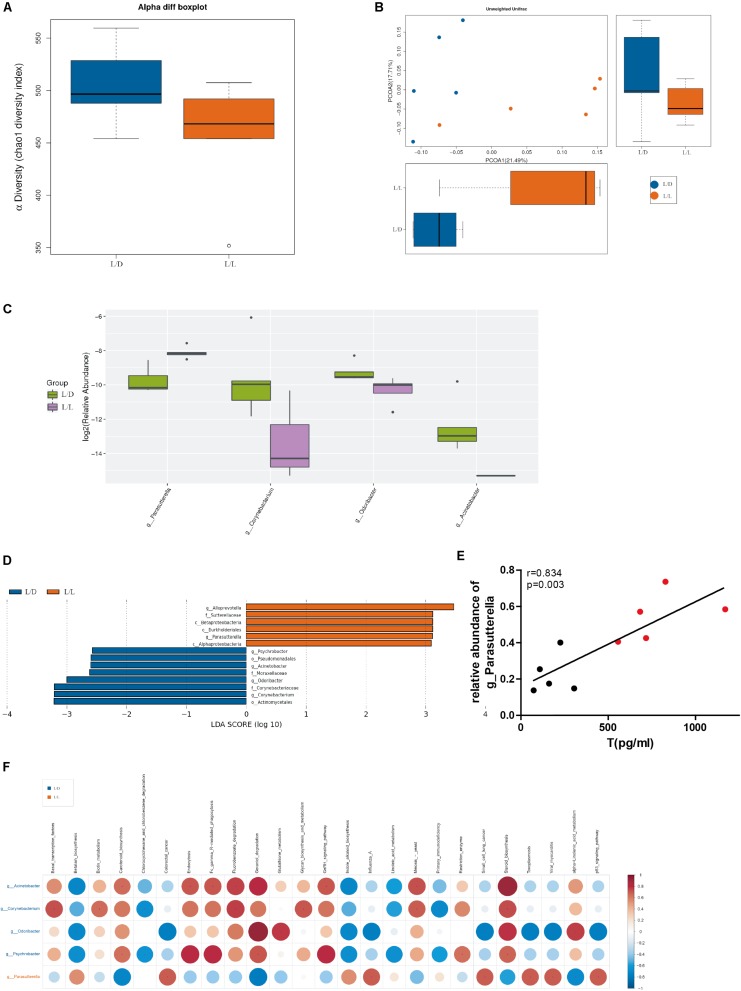
Gut microbiota was changed after continuous light exposure for 4 weeks. **(A)** Boxplots of α-diversity (Chao1 diversity index) between L/L and L/D groups at week 4. **(B)** PCoA plot with different relative abundances of OTUs between the groups at week 4. Difference between L/L and L/D groups was assessed by 2-way analysis of similarities (ANOSIM) analysis. Bacterial taxa significantly enriched in the L/L group compared to the L/D group were detected by LEfSe (*P* < 0.05, LDA > 2 logs). **(C)** The relative abundance in genus level difference indicated one taxa enriched in the L/L group (purple boxplot) and three taxa enriched in the L/D group (green boxplot). **(D)** Five taxa were enriched in the L/L group (red bars) while eight taxa were enriched in the L/D group (blue bars). **(E)** Correlation analysis between relative abundance of genus *Parasutterella* and serum testosterone level. **(F)** Prediction of functional potential with PICRUST analysis. Left axis indicated taxa enriched in the L/D (blue front) or L/L (red front) group and their predicted functional potential were displayed above. Red dots indicated a positive relationship, while blue dots in blue meant a negative relationship. *N* = 5/group. Significant differences between groups were indicated by *P* < 0.05.

Microbial community richness (alpha diversity) was measured by Chaol index. No significant difference was observed between L/D group and L/L group after 4 weeks of treatment (Figure 4A, *P* > 0.05). To examine beta diversity between L/D and L/L group of rats, unweighted Unifrac distance was calculated to estimate dissimilarities in the community membership. PCoA showed L/D and L/L group of rats harbored distinct microbial taxa (Figure 4B) and ANOVA (*R* = 0.42, *P* < 0.05) revealed significant differences in the community membership between L/L and L/D group of rats.

Additionally, the relative abundance analysis showed that some microbial taxa differed in significance between the L/D and L/L groups (Figure 4C). Continuous light exposure increased the abundance of *Parasutterella* spp., while it reduced that of *Corynebacterium* spp., *Odoribacter* spp., and *Acinetobacter* spp. Besides, LEfSe was employed to identify specific genera that differentially distributed between L/D and L/L group (Figure 4D). It showed that thirteen genera were distinctively represented between two groups, with six being more abundant in L/L group (e.g., *Alloprevotella* spp., *Sutterellaceae*, *Betaproteobacteria*, *Burkholderiales*, *Parasutterella* spp., and *Alphaproteobacteria*) and eight being abundant in L/D group (e.g., *Psychrobacter* spp., *Pseudomonadales*, *Acinetobacter* spp., *Moraxellaceae*, *Odoribacter* spp., *Corynebacteriaceae*, *Corynebacterium* spp., and *Actinomycetales*).

Through correlation analysis, we observed the relative abundance of *Parasutterella* spp. was in positive correlation with serum level of testosterone (Figure 4E, *r* = 0.834, *P* < 0.01). To infer the putative role of gut bacteria in the pathophysiological process, we performed PICRUSt analysis to predict the functional composition of a metagenome using marker gene data and a database of reference genomes (Figure 4F). Metagenomic inference indicated that L/L group of rats harbored microbiomes with a lower abundance of reproductive and metabolic-related genes, such as “gonadotropin-releasing hormone (GnRH) signaling,” “steroid biosynthesis,” and “glycan metabolism.” On the contrary, rats of L/D group had a greater abundance of these genes, suggesting a more stable and psychological role of gut flora in healthy rats. Gut microbiota profile shaped by continuous light exposure could be in close relevance with endocrine disturbance and glucose intolerance in PCOS.

## Discussion

PCOS is an important endocrine disorder with high prevalence in women of reproductive age. It can damage female fertility and increase the risk of metabolic disorders, such as obesity, insulin resistance, and type 2 diabetes (Johnstone et al., 2010; Bozdag et al., 2016). Genetics, environmental factors, epigenetic changes in fetal life, and hormonal imbalance all could contribute to PCOS, but the etiology of PCOS remains nebulous (Vink et al., 2006; Wu et al., 2010; Xu et al., 2011; Yu et al., 2015; Kokosar et al., 2016).

In the past few decades, much research has revealed the relationship between circadian rhythm disturbance and occurrence of PCOS. Sub-healthy lifestyle, including night-light shifts and transmeridian travels, is always accompanied by menstrual-related mood disturbances, abnormal menstrual cycles, and metabolic disorders, including insulin resistance and deregulation of glucose metabolism, all of which are considered risk factors for PCOS in reproductive age women (Chung et al., 2005; Baker and Driver, 2007). Since decisive clinical studies are limited by ethical and logistic constraints, a suitable animal model is an appropriate and valuable method to study the characteristic reproductive and metabolic abnormalities, clarify the relationship between circadian rhythm disturbance and PCOS, and reveal the intricate pathogenic progress underneath.

In our experiment, circadian rhythm disorder induced PCOS-like reproductive changes and glucose metabolism disorders in SD rats. After continuous light exposure for 4 weeks, the estrous cycle of rats was disrupted. The ovarian morphology in L/L group was similar with characteristic of PCOS, manifested as abundant atretic cyst-like follicles and reduced number of CLs, demonstrating oligo/anovulation. All these changes were in accordance with those of previous reports (Kang et al., 2015). Additionally, we found that AMH, the significant clinical parameter of PCOS women were remarkably increased compared to control group. For the first time we observed continuous light exposure could induce AMH elevation, in agreement with the clinical feature of PCOS patients (Dumont et al., 2015). It is well known that AMH can be used as a diagnostic and/or prognostic parameter in women in association with ovulation induction and in various pathophysiological conditions such as PCOS in clinical practice. The identical alteration in AMH level suggested without exogenous supplement of steroid hormones, light exposure played a vital role in developing AMH elevation, and circadian rhythm disruption might be indispensable in the pathological process of ovulation dysfunction in PCOS. Unchanged serum levels of corticosterone and TSH eliminated the effect of stress and strengthened the PCOS-like reproductive changes induced by continuous light exposure.

Additionally, we found impaired glucose metabolism in L/L group of rats, which was consistent with manifestation of PCOS in patients (Dumont et al., 2015; Łebkowska and Kowalska, 2017). Lower basal insulin level and HOMA-β indicated poorer β-cell secretion and function after continuous light exposure. The postprandial plasma glucose elevation might be attributed to poor β-cell function, as well. On the other hand, the altered gene expression in liver, adipose tissue, and muscle implies that insulin signaling is impaired owing to continuous light exposure. According to previous reports, insulin receptor, insulin receptor substrate 1/2, and glucose transporter type 4 are significant in glucose uptake and transportation progress physiologically (Abdul-Ghani and DeFronzo, 2010). After binding with IR, its membrane receptor, insulin triggers a signaling cascade to downstream substrates including IRS-1/2 and results in glucose transport. Some studies have found upregulated expression of IRS1, IRS2 in PCOS patients, and the circulating Leptin levels have been directly correlated with insulin resistance, which frequently is associated with PCOS (Dawood et al., 2018; He et al., 2019). Decreased abundance of IR and elevated expression of Leptin, IRS1, and Glut4 have confirmed that continuous light impairs glucose metabolism and contributes to the insulin resistance in PCOS rat models.

Alterations in gut microbiota induced by continuous light demonstrated the possible link between circadian rhythm disorder and PCOS-like phenotypes in reproduction and metabolism. It is well known that gut microbiota has an important role in energy metabolism (Velagapudi et al., 2010). Accumulating studies have indicated the relationship between imbalanced gut microbiota and clinical parameters in women with PCOS (Insenser et al., 2018; Torres et al., 2018). In our experiment, we analyzed the feces microbiota of rats with a 16S rRNA gene sequencing technique. In both L/D group and L/L group, the α-diversity and composition of their gut microbiota varied among different weeks. But the time-dependent *Firmicutes* accumulation as well as *Bacteroidetes* reduction in L/L group revealed the possible association between circadian disturbance and metabolic disorder in PCOS. Continuous light resulted in a higher Firmicutes/Bacteroidetes ratio (F/B ratio) in gut microbiota, which was rather similar to the transformation of metabolic disorder (Ferrocino et al., 2018; He et al., 2018). Former reports revealed administration of probiotics favorably influenced the microbiota with a decreased F/B ratio (Iqbal et al., 2018; Wang H. et al., 2018). Continuous light exposure might have an adverse impact on gut microbiota and glucose metabolism.

On the other hand, we found the microbiome was drastically differed between L/D group and L/L group of rats at the termination of experiment. The relative abundance of several bacterial taxa significantly altered after light exposure, with the enrichment of microbial genus *Parasutterella*, and the reduction of genus *Corynebacterium* and genus *Odoribacter*. Many scientists have discovered that increased *Parasutterella* is relevant with inflammation disease and immune system disorders (Huang et al., 2017; Chen et al., 2018; Wang X. et al., 2018). Some of them believed that the microbial genus *Parasutterella* is intimately associated with the inflammation reaction, altering gut motility and increasing gut wall permeability. In this respect, the imbalanced microbiota could possibly bring about inflammation in multiple organs and contribute to the pathologic progress of glucose intolerance in PCOS. In our experiment, the abundance of genus *Parasutterella* was positively correlated with serum testosterone level, suggesting the close relationship between *Parasutterella* and hyperandrogenism in PCOS. Moreover, through the PICRUSt analysis we found some predictive functional potential pathways of microbial genera. According to this analysis, the microbiome abundant in rats under continuous light exposure was negatively correlated to reproductive and metabolic-related genes, including “gonadotropin-releasing hormone (GnRH) signaling,” “steroid biosynthesis,” and “glycan metabolism,” which implied a possibly weaker and unbalanced state of microbiota in continuous light group. In this view, we assumed that there might be some intricate interactions between imbalanced gut microbiota, hormonal, and metabolic disorders. Gut microbiota probably served as a vital link between circadian rhythm disturbance and PCOS phenotype. Imbalanced gut flora shaped by continuous light could interfere with gut epithelial function and accelerate inflammation reaction, contributing to further insulin resistance and reproductive disturbance in PCOS. The specific role and mechanism of gut microbiota in this pathophysiologic process shall be investigated in the future.

In conclusion, we found circadian rhythm disorder could induce reproductive and endocrine system disruption, and glucose intolerance in SD rats. This study is to establish the impact of continuous light exposure on PCOS-like reproductive manifestation, as well as the diversity and composition of gut

microbiota. The altered gut microbiota demonstrated a possible link between circadian rhythm disorder and PCOS-related manifestations. Imbalanced gut microbiota caused by circadian disorder might contribute to the pathology progress of PCOS, while further experiments are required to explore the underlying mechanism. This study confirmed the role of circadian rhythm disorder in developing PCOS-like manifestations including reproductive hallmarks and glucose intolerance, and discovered the possible mechanism of PCOS pathogenesis that is enrolled with gut microbiota. It shed light on an investigation direction toward the pathologic mechanism of PCOS occurrence and provided basic research evidence for clinical diagnosis and treatment in reproductive women suffering from circadian rhythm disturbance.

## Data Availability Statement

The datasets generated for this study can be found in the NCBI Sequence Read Archive under Accession PRJNA508095.

## Ethics Statement

All experimental protocols were performed in accordance with institutional guidelines. The study was approved by the research committee of the Shanghai Jiao Tong University School of Medicine.

## Author Contributions

WC, JZ, JX, SL, and YD designed the study. WC and JZ contributed to conducting the experiments and analyzing the data. WC, JZ, SL, WL, Z-JC, and YD drafted and revised the manuscript. All authors reviewed the results and approved the final version of the manuscript.

## Conflict of Interest

The authors declare that the research was conducted in the absence of any commercial or financial relationships that could be construed as a potential conflict of interest.

## Footnotes

1 http://drive5.com/uparse/

2 http://rdp.cme.msu.edu/

3 http://rdp.cme.msu.edu/

## Supplementary Material

FIGURE S1 **(A–G)** The levels of LH, FSH, estradiol, progesterone, TSH, corticosterone, and leptin in serum of L/D and L/L groups of rats, respectively. **(H)** Histological sections of representative parametrial fat pads from L/D and L/L groups. *N* = 11/group. Values are expressed as means ± SEM.

TABLE S1 Primer Sequences.
