# Supplementary figures and images for: Continuous Light-Induced PCOS-Like Changes in Reproduction, Metabolism, and Gut Microbiota in Sprague-Dawley Rats
Source: Front Microbiol. 2020 Jan 21;10:3145. doi: 10.3389/fmicb.2019.03145 (PMC6990112; doi:10.3389/fmicb.2019.03145)

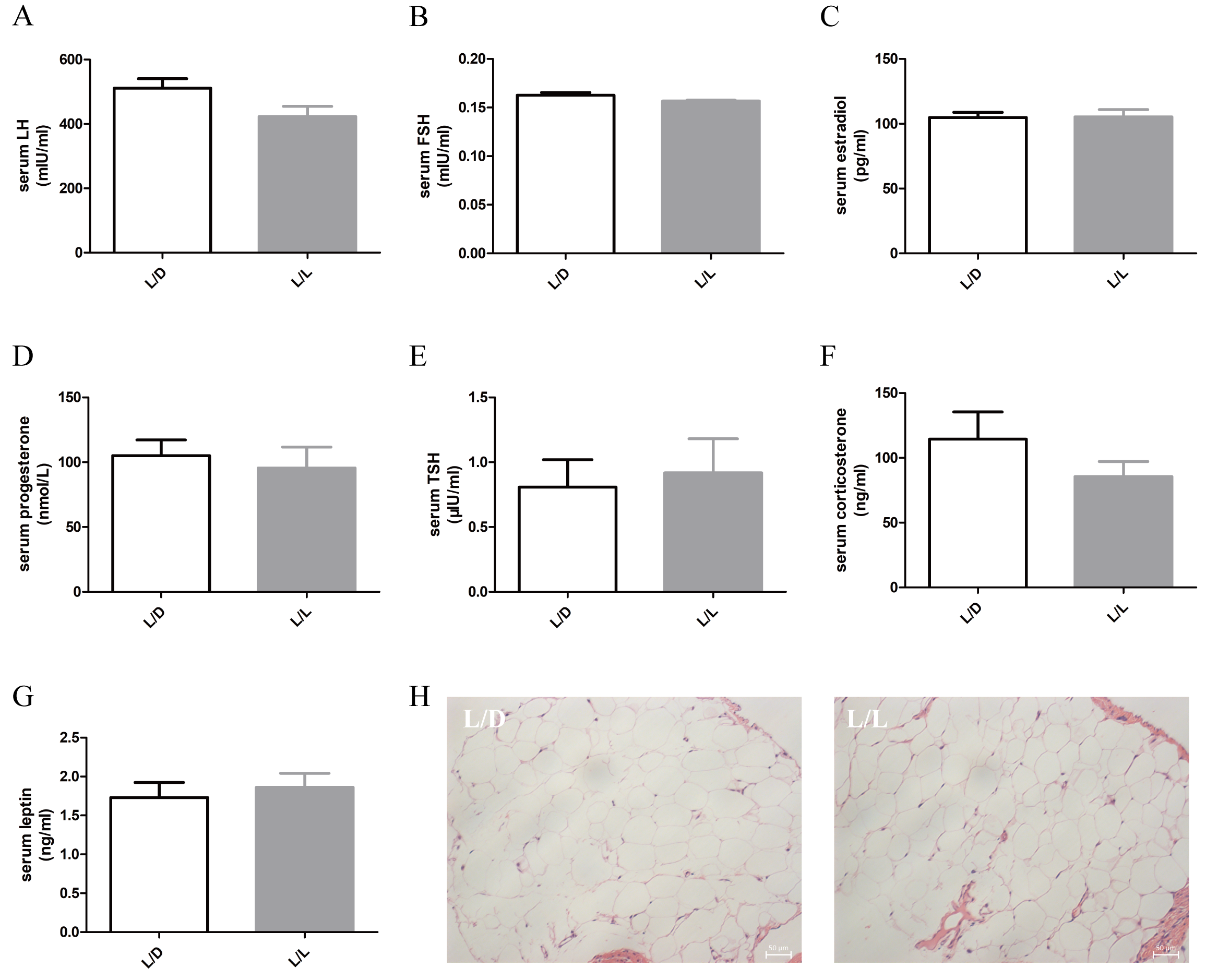

Supplement: FIGURE S1 — (A–G) The levels of LH, FSH, estradiol, progesterone, TSH, corticosterone, and leptin in serum of L/D and L/L groups of rats, respectively. (H) Histological sections of representative parametrial fat pads from L/D and L/L groups. N = 11/group. Values are expressed as means ± SEM. [file Image_1.TIF]
