# Supplementary material for: Continuous Light-Induced PCOS-Like Changes in Reproduction, Metabolism, and Gut Microbiota in Sprague-Dawley Rats
Source: Front Microbiol. 2020 Jan 21;10:3145. doi: 10.3389/fmicb.2019.03145 (PMC6990112; doi:10.3389/fmicb.2019.03145)
Supplement: TABLE S1 — Primer Sequences. [file Data_Sheet_1.docx]

**Supplementary Table 1. Primer Sequences**

| Target Gene | Forward sequences | Reverse sequence |
| --- | --- | --- |
| Leptin | ATCCTCACCAGCTTGCCTTC | CATCCAGGCTCTCTGGCTTC |
| Ir | TGAAGACCAGACCCGAAGATT | TCAAGACCAGACCCGAAGATT |
| Irs1 | TCTACACCCGAGACGAACACT | TGGGCCCTTTGCCCGATTATG |
| Glut4 | ACACTGGTCCTAGCTGTATTCT | CCAGCCACGTTGCATTGTA |
| β-actin | GGGAAATCGTGCGTGACATTAAG | TGTGTTGGCGTACAGGTCTTTG |
